# WHAT’S AGE, SEX, AND FRAILTY GOT TO DO WITH IT? USING DESIGN AND ANALYSIS TO PROVIDE POWERFUL ANSWERS

**DOI:** 10.1093/geroni/igae098.1647

**Published:** 2024-12-31

**Authors:** Heather Allore

**Affiliations:** Yale University, New Haven, Connecticut, United States

## Abstract

Designs of animal experiments to disentangle the heterogeneity associated with age, sex and frailty will be shared. These range from cross-sectional to longitudinal and cross-over designs (where treatment may be started and stopped). Matching the analytic models to the design should be determined a prior at the design stage. Considerations in longitudinal designs including within animal correlation across repeated measures. Hypotheses need to be written a priori clearly identifying the main effect and whether interactions between effects are potentially important, such as sex by frailty status. Batch effects of birth cohorts or caging may also need to be models as sources of variation to have the most accurate and precise effect estimates. Examples of such designs and analyses will be presented, as well as sample size considerations. Properly designed animal experiments area key element in translational gerontology and geroscience. Planning for the factors in animal experiments that are known or suspected risk factors for geriatric outcomes in humans allows for testing whether the animal model may appropriately model the human conditions under study.

